# Laparoscopic Management of Interstitial Pregnancy and Fertility Outcomes after Ipsilateral Salpingectomy – Three Case Reports

**DOI:** 10.3389/fsurg.2014.00034

**Published:** 2014-09-05

**Authors:** Cristina Manea, Evangelia Pavlidou, Aline Andrey Urias, Jean Bouquet de la Jolinière, Jean Bernard Dubuisson, Anis Feki

**Affiliations:** ^1^Department of Obstetrics and Gynecology, Hôpital Cantonal Fribourgeois, Fribourg, Switzerland; ^2^Department of Obstetrics and Gynecology, Geneva University Hospitals, Geneva, Switzerland

**Keywords:** interstitial pregnancy, laparoscopic treatment, minimally invasive laparoscopy, ectopic pregnancy, methotrexate, ipsilateral salpingectomy

## Abstract

**Background:** Interstitial pregnancy after ipsilateral salpingectomy is a rare event with potentially serious consequences. Optimal management strategy remains uncertain and debated. In addition, fertility sparing is determinant of the treatment choice.

**Cases:** Here, we report three cases of interstitial pregnancy occurring after homolateral salpingectomy. We expose the therapeutic option held in all three situations, which associated laparoscopic procedure followed by intramuscular methotrexate injection with successful outcome for all patients. We also report the fertility outcome for the first patient, discussing the timing and mode of delivery. Cesarean section at term was performed for this patient.

**Conclusion:** In these three situations, we obtained a successful result using a minimally invasive surgical approach combined with systemic methotrexate injection. Cesarean section at term for subsequent intrauterine pregnancy seems to be the safest delivery strategy, although no clear data exist in literature.

## Introduction

One of the main causes of maternal mortality is the extrauterine pregnancy (1). The interstitial, cornual, and angular pregnancies are often grouped together, despite the fact that their behavior, management, and outcome are different, thus causing confusion in clinical practice. In this article, we will focus on the ectopic pregnancy localized in the interstitial portion of the fallopian tube.

Interstitial pregnancy refers to an ectopic pregnancy that is implanted in the interstitial portion of the fallopian tube, which is defined as the tubal segment traversing the muscular wall of the uterus (2–4). This term also includes the cases of the development of trophoblastic tissue in the remaining tubal part following salpingectomy (5).

Interstitial pregnancy accounts for 1–6% of all ectopic pregnancies (6) and 2–4% of all tubal gestations (2). Unfortunately, the mortality rate of interstitial pregnancy still remains at 2–2.5% (2). The main risk factor for interstitial pregnancy is the antecedent of an ipsilateral salpingectomy (7). Other risk factors for ectopic pregnancy include past pelvic inflammatory disease and salpingitis isthmica nodosa, previous pelvic surgery, and uterine anomalies. Furthermore, the frequency of interstitial implantation has increased from 1.9 to 7.3% by the use of assisted reproductive techniques (8).

Patient’s gestational age, hemodynamic condition, and desire for future pregnancies, as well as the medical center’s surgical assets will influence the treatment approach. The surgical option is often chosen for the management of the interstitial pregnancy since at the time of the diagnosis the rupture of the gestational sac is a frequent finding. Nevertheless, in case of early diagnosis with intact gestational sac and hemodynamically stable patients, it is possible to follow a conservative treatment with local or systemic administration of methotrexate (9). Other drugs such as potassium chloride or etoposide (10) have also been used successfully.

Taking into account the progress on the equipment and the expertise in the laparoscopic field during the last years, laparoscopy has justifiably become the most preferable method (4), even replacing laparotomy in certain cases of hemodynamic instability.

This paper reports three cases of laparoscopic therapeutic procedure on ruptured interstitial pregnancies, after prior homolateral salpingectomy.

## Patient History

### Case 1

A 21-year-old woman (gravida 1, para 0) arrived at the emergency department with mild suprapubic pain, described as “menstrual cramps” at 7 weeks of amenorrhea. She had stopped the contraceptive pill some months before without planning on having children immediately. Her medical history included smoking (half a packet per day), asthma, tonsillectomy, and a single incision laparoscopic surgery (SILS) with right salpingectomy for the torsion of a right paratubal cyst 6 months earlier. She was in pain but conscious and well oriented. Her vital signs were normal. The abdominal palpation was painful in the right iliac fossa but without guarding or rebound tenderness. Other physical examination findings were normal with the exception of minimal “spotting” bleeding. The urinary pregnancy test was positive, the beta human chorionic gonadotrophin (β-hCG) was 1574 U/L, and the progesterone was 12.8 μg/L. The ultrasonographic (US) examination revealed: a normal-volume anteverted uterus with an endometrial thickness of 16 mm and a hypoechoic intrauterine image of 3 mm in diameter, compatible with an eventual gestational sac, without any adnexal mass or intra-abdominal fluid. The patient was re-examined 48 h later. At that time, the clinical examination and ultrasound findings were identical; the β-HCG levels increased at 2238 U/L and the patient was discharged from the hospital with a follow-up appointment in 1 week.

However, 72 h after the first examination, the patient returned to the hospital with acute abdominal pain, which appeared suddenly during the night. The vital signs were: blood pressure 113/80 mm Hg, heart rate 88 beats/min, temperature 37.5°C, and oxygen saturation 97%. On abdominal palpation, she was tense and distended, with guarding and rebound tenderness in the right iliac fossa. Gynecologic examination revealed discrete vaginal bleeding and a painful bimanual pelvic exam. The patient’s hemoglobin level, white blood cell count, and platelet count on admission were 106, 8.8, and 250 g/L, respectively. The US revealed a uterine interstitial subserosal mass of 17 mm × 14 mm and a hypoechogenic image of 15 mm × 15 mm compatible with fluid in the pouch of Douglas. The patient underwent a diagnostic laparoscopy after a detailed informed consent was signed.

**Figure 1:**
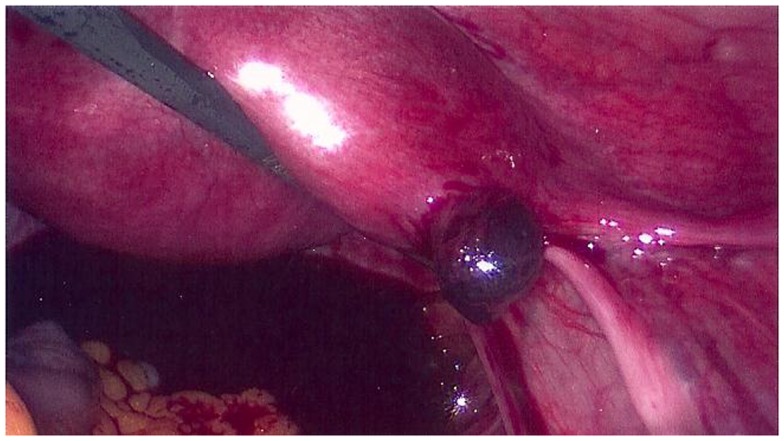
**Right interstitial pregnancy after ipsilateral salpingectomy (case 1)**.

Laparoscopic findings established a diagnosis of a ruptured right interstitial pregnancy with a hemoperitoneum of 100 mL (Figure 1). The product of gestation was removed by Johan grasping forceps, followed by a remnant right tube expression (“milking” procedure). The hemostasis of the implantation area was performed by bipolar coagulation and the uterine cornu was closed by two single “X” absorbable sutures.

### Case 2

A 38-year-old woman (gravida 4, para 1) arrived at the emergency department with an acute suprapubic pain, worsening at mobilization, and 5 weeks of amenorrhea. Her medical history included a normal delivery at 34 weeks 10 years ago, a laparoscopy with right salpingotomy for an ectopic pregnancy 4 years earlier, and a SILS with right salpingectomy for another extrauterine pregnancy 3 months earlier. On initial physical examination, her vital signs were: blood pressure 112/70 mm Hg, heart rate 83 beats/min, and temperature 37.3°C. She was in pain, but conscious and well oriented. The abdominal palpation was normal with no guarding or rebound tenderness, while the bimanual pelvic exam revealed uterine tenderness, without adnexal tenderness or mass. The patient’s hemoglobin level, white blood cell count, and platelet count on admission were 124, 8.2, and 206 g/L, respectively. On admission, the level of β-HCG was at 6892 U/L and the progesterone at 21.1 μg/L. The US examination revealed a right retrouterine mass, compatible with a ruptured extrauterine pregnancy, and a hemoperitoneum of about 200 mL. The patient underwent a diagnostic laparoscopy after a detailed informed consent was signed.

Laparoscopic findings established a diagnosis of a ruptured right interstitial pregnancy with a hemoperitoneum of 600 mL. After removal of the gestation product by Johan grasping forceps, injection of a diluted solution of 0.7 mg of adrenaline circumferentially into the myometrium beneath and lateral to the pregnancy was realized in order to minimize local bleeding. A corneal wedge resection was then performed using a laparoscopic monopolar needle. After washing and checking the incision site, the myometrium was sutured using two single “X” stitches of 2.0 absorbable threads. The abdominopelvic cavity was then cleaned with Lactated Ringer’s solution.

### Case 3

A 35-year-old woman (gravida 6, para 1) presented at the ER with acute abdominal pain 1 month after *in vitro* fertilization procedure with a frozen embryo transfer (IVF-ET). Her vital signs were stable apart from decreased blood pressure of 94/52 mm Hg without tachycardia. Her past obstetric and gynecologic history included one previous spontaneous birth, one missed pregnancy, and three ectopic pregnancies for which she underwent bilateral salpingectomy. She was at the time treated for secondary sterility for 3 years. On physical examination, the patient’s vital signs were stable and she had abdominal guarding and diffuse rebound tenderness. A transvaginal ultrasound scan revealed a left adnexal mass near the left uterine corner and a small intrauterine hypoechogenicity and little free fluid in the pelvis. Blood counts were as follows: hemoglobin: 115 g/L; WBC: 4.7 g/L; platelets: 356 g/L. Her β-HCG level was 8915 U/L and the progesterone was 17.3 μg/L.

**Figure 2:**
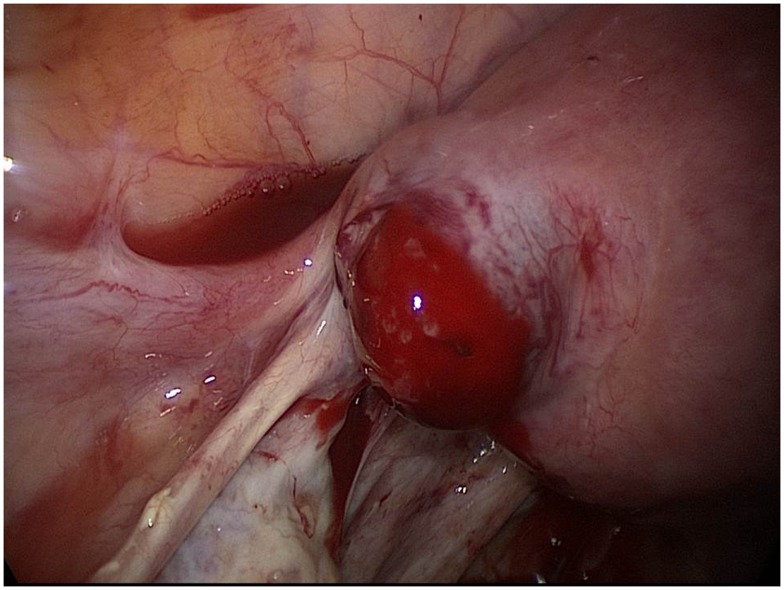
**Left interstitial pregnancy after ipsilateral salpingectomy (case 3)**.

**Figure 3:**
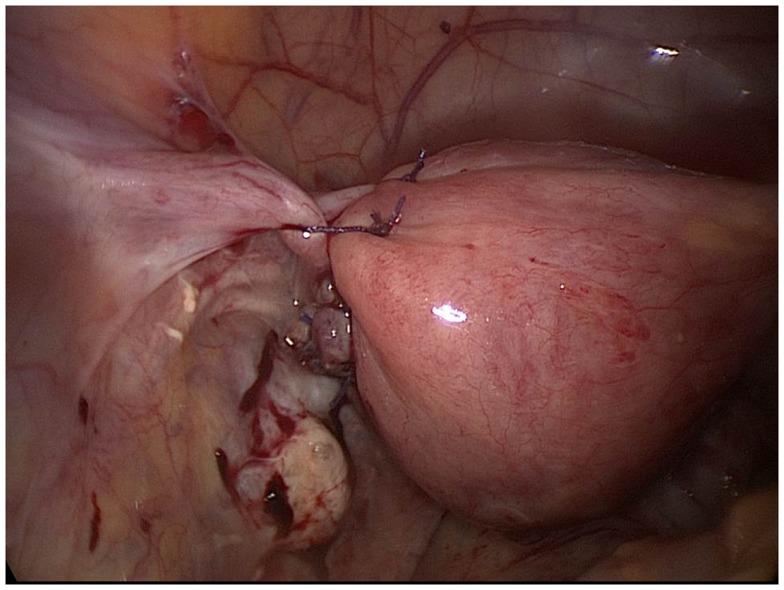
**Final result of surgery in case 3**.

Laparoscopic findings established a diagnosis of a ruptured left interstitial pregnancy (Figure 2). Free peritoneal blood was removed by suction (about 600 mL). The tubal wall covered by myometrium was incised with laparoscopic monopolar electrode and the product of gestation removed with grasping forceps and sent to pathology. Hemostasis was maintained by bipolar coagulation. The left uterine horn was closed by three “X” sutures and reinforced furthermore by stitching the round ligament around the stump (Figure 3). The same procedure was used on the opposite side to allow a median posture of the uterus. A uterine curettage was conducted at the end of operation. The pathology confirmed the presence of trophoblastic cells in the tubal stump and that of the curettage showed images of Arias Stellas.

### Treatment outcome

The average operative time was 1 h. The blood loss was minimal for the first patient, while for the second one it was estimated at 900 mL (hemoperitoneum of 600 mL and perioperative blood loss of 300 mL) a hemoglobin control was performed 6 h after the operation, revealing an anemia with a hemoglobin level at 93 g/L. The third case showed similar blood loss of 600 mL with postoperative hemoglobin of 88 g/L. Intramuscular methotrexate administration (1 mg/kg) was performed for all three patients in order to ensure complete regression of the trophoblastic tissue. The postoperative course was uneventful in all cases and they were discharged from the hospital on the first postoperative day. The patients were followed up with β-HCG measurement on a weekly basis, until the β-HCG level decreased below 5 U/L (23 and 29 days postoperatively for the first and the second case, respectively). The histological examination confirmed the extrauterine pregnancy. All patients were advised to use an estrogen–progestin contraceptive for at least 6 months after the operation.

Despite medical counseling to avoid pregnancy for at least 6 months, a new pregnancy occurred 2 months after the treatment of the ectopic pregnancy in the first patient. This intrauterine pregnancy was carried out successfully and a cesarean section was performed, resulting in the birth of a healthy child.

## Discussion

The interstitial pregnancy remains one of the rarest types of ectopic pregnancy. With the development of assisted reproduction techniques, an increase in the incidence of such pregnancies could be expected. However, the cases of spontaneous interstitial pregnancy after prior homolateral salpingectomy reported in the literature are still very few.

The risk factors for the interstitial pregnancy are the same as for the other types of ectopic pregnancy (tubal damage from previous ectopic pregnancy, pelvic inflammatory disease, the use of the assisted reproductive technology, prior pelvic operation, tumors, and uterine anomalies), with the exception of ipsilateral salpingectomy, which remains a risk factor unique to interstitial pregnancy (2, 11).

As far as the treatment of the interstitial pregnancy is concerned, the conservative methods (i.e., methotrexate) as well as several surgical procedures have been reported. The fact that the blood supply to the region of the intramural tubal segment comes from both uterine and ovarian arteries makes the rupture of an interstitial pregnancy a high-risk incident, often complicated by a severe hemorrhage and higher maternal morbidity and mortality rates than other ectopic pregnancies (9, 12). As a result, the conservative method of methotrexate can be attempted only in cases of hemodynamically stable patients with an early stage diagnosis. Certainly, by avoiding the surgical procedure the probability of preserving fertility is increased (12). Local or systemic administration of methotrexate seems to be effective, with an overall success rate of 83% (2). Some studies suggest that direct corneal methotrexate application might carry higher success rates (13). Other medical treatments showing some success include both etoposide and potassium chloride, both injected directly into the gestational sac under US visualization (14). There has been a report of a heterotopic interstitial and intrauterine pregnancy where the ectopic pregnancy was resolved by injecting (under sonographic guidance) potassium chloride directly into the embryo (15). Similarly, hysteroscopic suction may be attempted, especially if the uterine ostium is dilated (16).

Surgical approach includes the more traditional cornual excision by laparotomy, as well as the newer hysteroscopic, laparoscopic, and minimally invasive techniques, while several combinations of the different therapeutic methods have also been reported. Some years ago, cornual excision by laparotomy was the method of choice for the interstitial pregnancies with a small gestational sac and supravaginal hysterectomy was performed in the more advanced pregnancies (17). However, laparotomy was gradually replaced by laparoscopic techniques because of the advantages in terms of reduced hospitalization, faster recovery, and lower health costs (18). Cornual wedge resection, cornuostomy, mini-cornual excision salpingectomy, placing a Vicryl loop on the uterine cornu and salpingotomy are the laparoscopic techniques most frequently reported in the literature, with or without adjuvant methotrexate injection after surgery (4, 19–22). It should be noted that the Vicryl loop carries the risk to be placed laterally to the pregnancy (20) and it tends to slip (22), resulting in the failure of the pregnancy’s termination and demanding post-surgical supplementary interventions. The technique of aspiration in order to remove out the gestational products is preferable, since their removal gradually with grasping forceps or aspiration may be incomplete (2). In all cases of interstitial rupture, interstitial salpingotomy, and cornual wedge resection, it is necessary to close the uterine horn with absorbable sutures. Alternative surgical methods include the use of laparoendoscopic single-site surgery (23), the use of a temporary tourniquet suture in order to reduce bleeding (24), the laparoscopic salpingocentesis with methotrexate in combination with oral mifepristone (25), and the laparoscopic and ultrasound-guided transcervical evacuation (26). In order to avoid tubal rupture, the possibility of leaving the placenta *in situ* during transcervical evacuation of the gestational products under abdominal US guidance has also been reported, under the condition that an IM dose of methotrexate should be administered immediately after the operation (27). There are few cases treated by hysteroscopic management under sonographic guidance, or combination of laparoscopy, hysteroscopy, and ultrasound (28, 29). Finally, hysterectomy is strictly limited to the cases of uncontrollable hemorrhage, very large interstitial pregnancies, co-existing uterine pathology indicating a hysterectomy or women without fertility desire (4).

Regarding fertility, no differences in terms of pregnancy rates were established between the alternative treatment strategies in tubal ectopic pregnancies, although only limited information is available in literature regarding the specific cases of interstitial pregnancies (30, 31) (Table 1). Due to the risk of uterine rupture after an interstitial pregnancy, any subsequent pregnancy should be monitored carefully, and an elective cesarean delivery at term should be preconized to avoid the risk of uterine rupture during labor (32).

**Table 1:** **Reported interstitial pregnancy management and fertility outcome**.

Reference	No. of cases	Management			Fertility	Notes
		MTX i.m	MTX local	Surgical management	
Jermy et al. (2004) (6)	20	17	-	2 cornual resection by laparotomy	N.A	4 cases with fetal heart activity present (100% success) – 2 cases with 2 doses MTX
Surbone et al. (2013) (13)	11	3	6*4 during LSC; 2 transvaginal	2 LSC cornual resection	9 pregnancies	6 c-section at term
Selma et al. (2009) (19)	53	9 MTX Post-surgical		53 LSC; 33 wedge resection; 13 cornuostomy; 7 salpingectomy	18 pregnancies	5 vaginal deliveries, 3 c-sections
Soriano et al. (2008) (20)	27	3	1 hysteroscopic-guided injection in amniotic sac	20 Wedge resection and/or Vycril loop placement	NA	1 case managed with transvaginal sonography-guided KCl injection to the amniotic sac
Siow et al. (2011) (21)	4	2 MTX post-surgical		4 LSC wedge resection after vassopresin injection, repair with mattress sutures	3 pregnancies	2 c-sections at term and 1 vaginal delivery at 36 gestational weeks
Lazard et al. (2011) (23)	2			2 SILS cornual resection with automatic stapler	NA	SILS-single incision laparoscopic system
Young-Sam et al. (2009) (24)	8			8 LSC cornuostomy using a tourniquet suture and vasopressin inj	NA	
Latika et al. (2009) (25)	2			2 LSC salpingocentesis with MTX (50 mg/kg) after aspiration of an equivalent amount of amniotic fluid + 200 mg myfepristone p.o.	1	1 c-section at term
Pluchino et al. (2009) (33)	1			LSC cornuostomy and hemostatic suture	NA	
Moon et al. (2000) (34)	24	1 MTX postoperative		24 LSC; 15 Endoloop before evacuation of conceptus; 3 Encircling suture; vasopressin injection and electric coagulation	3 missed abortion; 1 ectopic pregnancy; 11 term pregnancies	11 c-sections at term
Tinelli et al. (2010) (14)	3	3 MTX postoperative		3LSC cornuostomy, hemostasis with bipolar forceps and suture with 4 single, U, sutures of 0 absorbable monofilament	NA	
Simpson et al. (1961) (7)	6			3 laparotomies cornual resection; 3 total abdominal hysterectomies	4 pregnancies	4 deliveries at term

Interestingly, there is a lack of data regarding the optimal interval to conception following surgical treatment of an ectopic pregnancy. In our practice, we advise patients to wait approximately 6 months before trying to conceive in order to minimize the risk of uterine rupture.

## Closing Remarks

By the three cases reported here, we confirm that laparoscopy is feasible and can be effective methods for the management of interstitial pregnancies, even when ruptured, in hemodynamically stable patients (33, 35, 36). We believe that for small ectopic pregnancies, in stable patients with low HCG levels a single dose of systemic MTX can be an effective option.

As there are no sufficient data available regarding the ideal method of treating interstitial pregnancies, the individualized approach in line with the clinician’s expertise as well as the patient’s clinical features and fertility desire should finally determine the choice of therapeutic procedure acceptable for both parties.

## Conflict of Interest Statement

The authors declare that the research was conducted in the absence of any commercial or financial relationships that could be construed as a potential conflict of interest.

## Supplementary Material

The Supplementary Material for this article can be found online at http://www.frontiersin.org/Journal/10.3389/fsurg.2014.00034/abstract
